# Tocilizumab in the treatment of IgG4-related disease involving the carotid artery-case report and literature review

**DOI:** 10.3389/fimmu.2026.1734761

**Published:** 2026-03-06

**Authors:** Xiaoxu Ding, Juan Tao, Cheng Xu, Yongmei Han

**Affiliations:** 1 Department of Rheumatology, Sir Run Run Shaw Hospital, Zhejiang University School of Medicine, Hangzhou, Zhejiang, China; 2 Nursing Department, Sir Run Run Shaw Hospital, Zhejiang University School of Medicine, Hangzhou, Zhejiang, China

**Keywords:** carotid artery, glucocorticoids, IgG4-related disease, tocilizumab, vagus nerve

## Abstract

IgG4-related disease (IgG4-RD) is an autoimmune disorder characterized by fibroinflammatory infiltration of affected organs. This systemic condition can involve multiple organs, including the pancreas, bile ducts, salivary glands, kidneys and lungs. However, IgG4-related disease demonstrates substantial heterogeneity and frequently involves atypical anatomical sites, leading to frequent clinical misdiagnosis. We report a rare case of IgG4-RD involving the carotid artery and vagus nerve, presenting with hoarseness and syncope. Diagnosis was confirmed via contrast-enhanced neck MRI and carotid artery biopsy. The patient exhibited poor response to glucocorticoids and methotrexate but achieved sustained remission following combination therapy with tocilizumab. A literature review revealed that isolated involvement of the carotid artery and vagus nerve in IgG4-RD is exceptionally rare.

## Introduction

IgG4-related disease (IgG4-RD) is an autoimmune disorder characterized by fibroinflammatory infiltration of affected organs, typically presenting as tumor-like masses. The pathological hallmark is characterized by dense infiltrates of IgG4-positive plasma cells (1). This condition demonstrates multiorgan involvement, commonly affecting the pancreas, bile ducts, salivary glands, kidneys, and lungs (2). Historically described diseases including Mikulicz’s disease and Riedel’s thyroiditis have subsequently been recognized as part of the IgG4-RD spectrum (3). Atypical clinical presentations may occur when IgG4-RD involves uncommon anatomical sites such as the testes, inner ear, or gastroduodenal region (4–6). Diagnosis of these rare-site manifestations proves particularly challenging, frequently resulting in significant delays between symptom onset and definitive diagnosis. Glucocorticoids currently serve as the cornerstone of IgG4-RD treatment, with immunosuppressive agents being added in cases of severe disease or inadequate response to steroids. Biological agents may be further incorporated into the therapeutic regimen when indicated (7). We herein present an exceptionally rare case of IgG4-RD involving both the carotid artery and vagus nerve, expanding the recognized phenotypic spectrum of this protean disease.

## Case presentation

**Figure 1:**
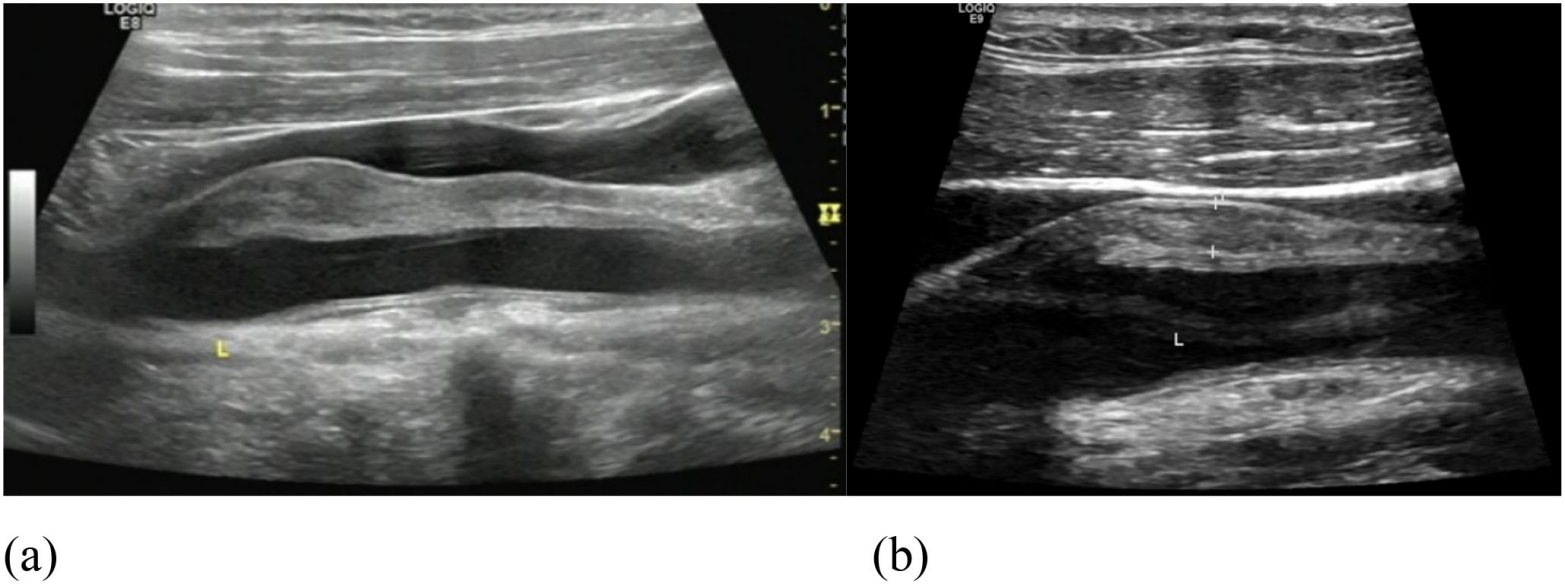
**(a)** Ultrasound before treatment showed a hypoechoic mass around the left common carotid artery. **(b)** Post-treatment ultrasound demonstrated reduction in the size of the hypoechoic mass surrounding the left common carotid artery.

**Figure 2:**
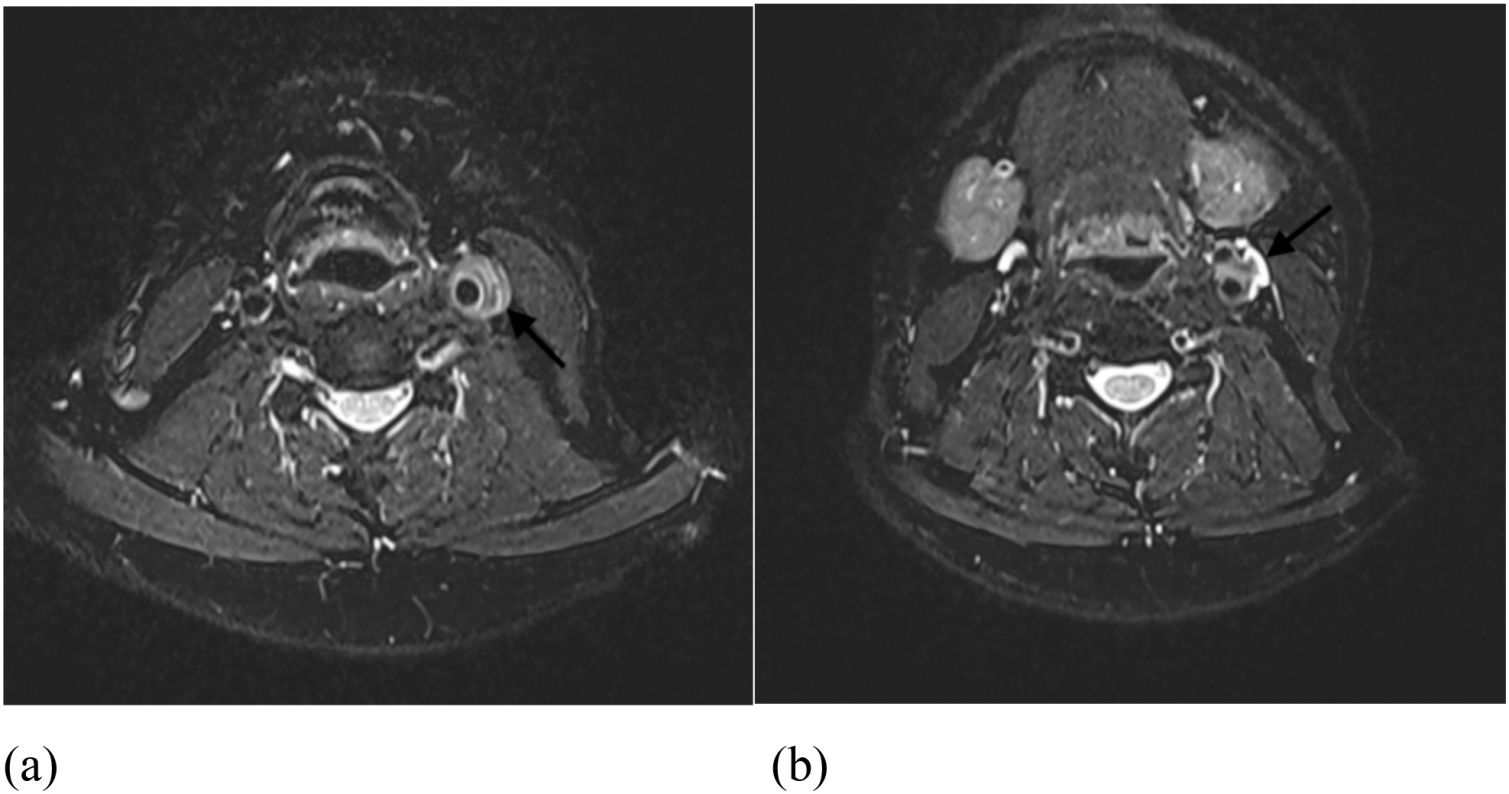
Contrast-enhanced neck MRI demonstrates: **(a)** Pericarotid mass lesions on T2-weighted sequences; **(b)** Mass lesions surrounding the carotid sinus on T2-weighted sequences.

**Figure 3:**
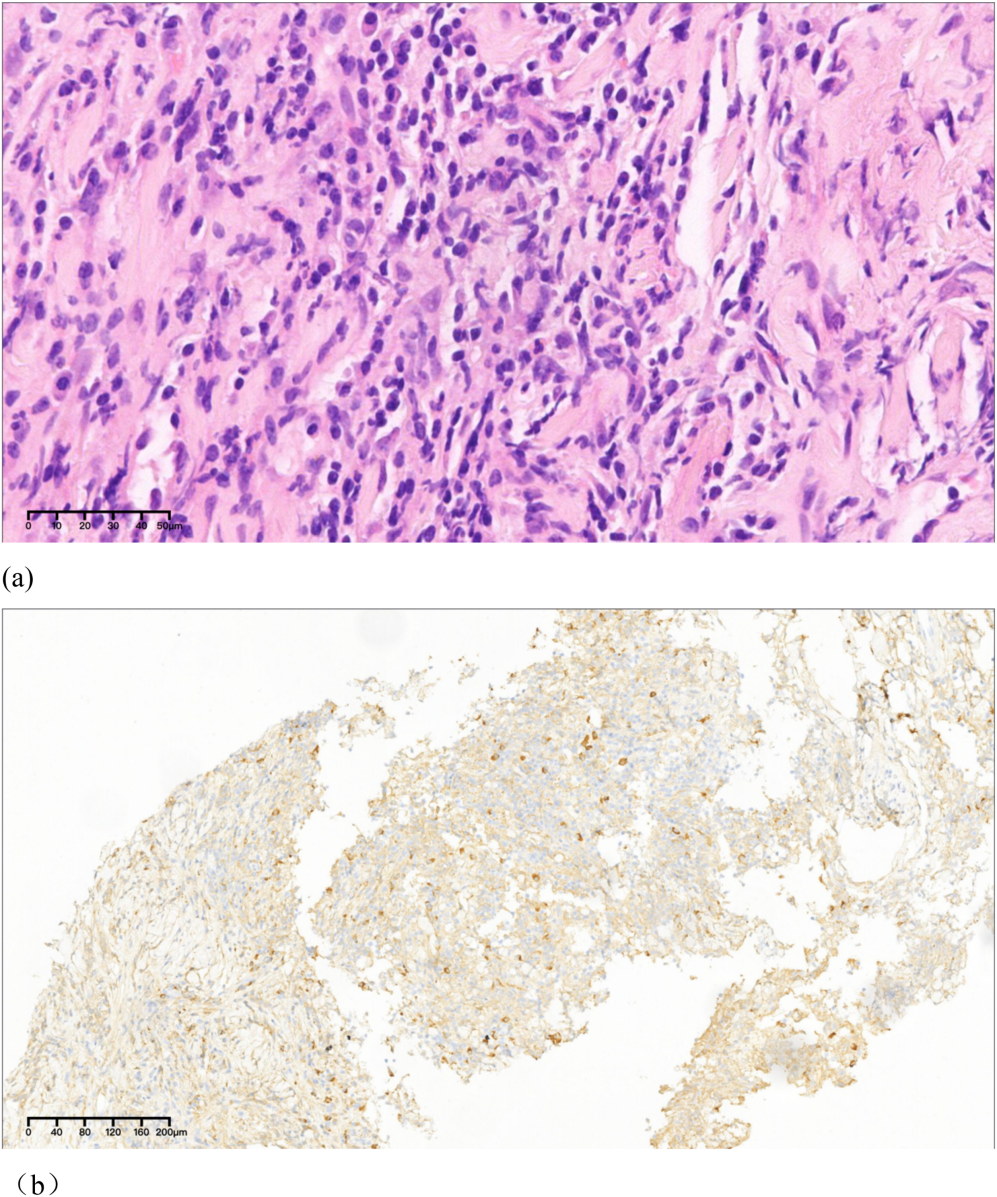
Histopathological and immunohistochemical findings of the left carotid artery wall biopsy. **(a)** Histopathological examination revealed fibrotic tissue hyperplasia with dense lymphocytic infiltration. **(b)** Immunohistochemistry demonstrated 12 IgG4−positive plasma cells per high−power field and an IgG4+/IgG+ plasma cell ratio slightly above 40%. Storiform fibrosis and obliterative phlebitis were absent. Sparse eosinophils were noted, while granulomas, prominent neutrophilic inflammation, necrosis, or other features suggestive of alternative diseases were not observed. Immunohistochemical staining was performed using a rabbit anti−human IgG4 antibody (clone EP138) and an IgG antibody (clone GR326) via the EnVision detection system.

A 41-year-old male presented with recurrent syncope and hoarseness. Three months prior, he experienced sudden syncope with documented hypotension (63/37 mmHg) and bradycardia (52 beats/min), which resolved after epinephrine administration. One week later, he was readmitted for recurrent syncope with undetectable blood pressure, again responsive to epinephrine treatment. Subsequently, he developed persistent syncopal episodes and hoarseness. His medical history included hypertension managed with long-term antihypertensive therapy. Family history was unremarkable, with no history of smoking or alcohol use. Physical examination revealed left cervical tenderness, prompting consideration of thyroid disorders, lymphoma, or Takayasu arteritis. Laboratory tests showed slightly elevated serum IgG4 (2.04 g/L; reference range: 0.03-2.01 g/L) with negative autoantibodies (ANA, RF, ANCA). ESR and CRP were within normal limits. Complete blood count, tumor markers, and thyroid function tests showed no abnormalities. Liver and kidney function were essentially normal. Ultrasound demonstrated hypoechoic perivascular tissue surrounding the left common carotid artery extending to the internal/external carotid bifurcation, encasing the swollen vagus nerve (**Figure 1a**). Contrast-enhanced MRI revealed circumferential wall thickening and enhancement of the left carotid artery, suggestive of vasculitis (**Figure 2**). To characterize the mass, an ultrasound-guided core needle biopsy of the left pericarotid lesion was performed, revealing histopathological features of fibrotic tissue hyperplasia with dense lymphocytic infiltration. Immunohistochemistry demonstrated 12 IgG4−positive plasma cells per high−power field (HPF), with an IgG4+/IgG+ plasma cell ratio slightly above 40%. Histopathological examination revealed no storiform fibrosis or obliterative phlebitis. Sparse eosinophils were present, while granulomas, prominent neutrophilic inflammation, necrosis, or other features suggestive of alternative diseases were absent (**Figure 3**). According to the 2019 American College of Rheumatology/European Alliance of Associations for Rheumatology (ACR/EULAR) classification criteria for IgG4−RD, the pathology showed dense lymphoplasmacytic infiltration, an IgG4+/IgG+ plasma cell ratio slightly above 40%, 12 IgG4+ plasma cells per HPF, a serum IgG4 level <2 times the upper limit of normal, yielding a cumulative weighted score of 22. Based on the 2020 Revised Comprehensive Diagnostic (RCD) criteria, the patient presented with a left cervical mass encasing the carotid artery and vagus nerve, elevated serum IgG4 (>135 mg/dL), histopathology showing dense lymphoplasmacytic infiltration with fibrosis, an IgG4+/IgG+ plasma cell ratio slightly above 40%, and 12 IgG4+ plasma cells per HPF. PET−CT and biopsy results revealed no evidence of malignancy. Thyroid function, ESR, and CRP were all normal, excluding lymphoma, thyroid disorders, and Takayasu arteritis from the diagnosis. In summary, the final diagnosis was IgG4−related disease involving the carotid artery and vagus nerve. Systematic assessment for IgG4-RD revealed no additional organ involvement.

**Figure 4:**
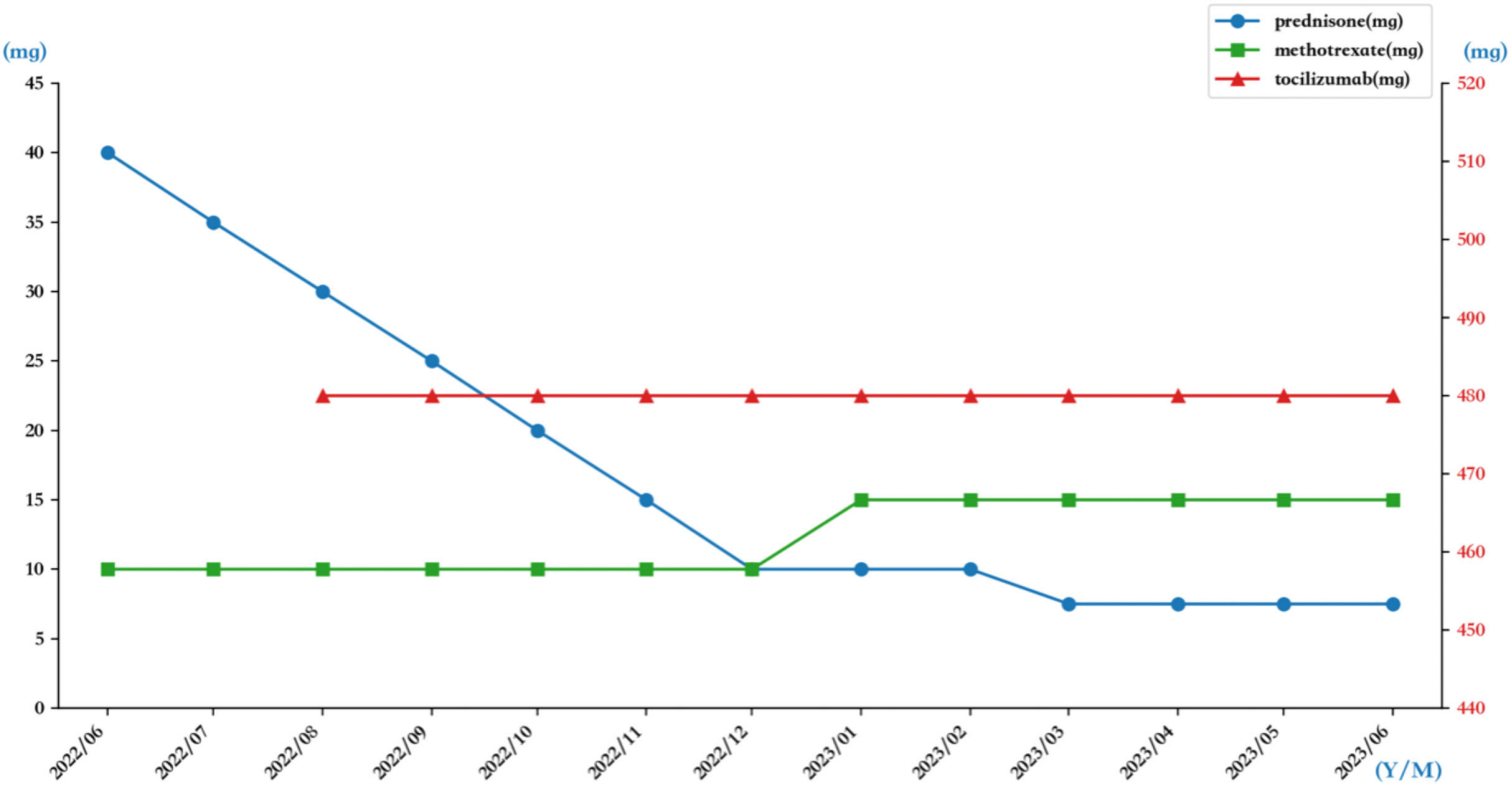
The medication use of prednisone, methotrexate, and tocilizumab during the treatment process.

The patient was treated with prednisone 40mg daily (qd) and methotrexate 10mg weekly (qw), with gradual tapering of steroids. Follow-up at 2 months revealed suboptimal therapeutic response: although serum IgG4 levels had normalized, ultrasound evaluation showed no reduction in the size of the left neck mass, and clinical symptoms such as hoarseness showed no improvement. Subsequent administration of tocilizumab 480 mg every 4 weeks (8 mg/kg, q4w) resulted in significant improvement in hoarseness and other clinical symptoms. After 12 months of follow−up, during which glucocorticoid dosing was progressively tapered, the patient was maintained on a regimen of prednisone 7.5 mg qd, methotrexate 15 mg qw, and tocilizumab 480 mg q4w (**Figure 4**). Ultrasound examination demonstrated reduction in the size of the left cervical mass (**Figure 1b**). Currently, the prednisone dose has been further tapered to 2.5 mg every other day. No significant treatment−related complications have been observed.

## Discussion

This report describes the first documented case of IgG4-RD with isolated involvement of both the carotid artery and vagus nerve, presenting with syncope and hoarseness as initial symptoms. While carotid artery involvement in IgG4-RD has been previously reported - including a 67-year-old man with motor aphasia and limb dysfunction found to have left internal carotid artery stenosis accompanied by carotid dissection and intracranial/cervical pseudoaneurysms (diagnosed via vascular biopsy) (8), a 69-year-old woman with intermittent headaches showing a parapharyngeal mass encasing the internal carotid artery and vein (9), and a 74-year-old woman with uniform thickening of the left carotid artery wall diagnosed with IgG4-related carotid arteritis (10).Our patient’s syncope likely resulted from mass compression of the carotid sinus, while hoarseness may be attributed to vagus nerve swelling affecting its branches - the recurrent laryngeal nerve and superior laryngeal nerve. Reports of vagus nerve involvement in IgG4-RD remain scarce. In 2019, a Japanese case of IgG4-related hypertrophic pachymeningitis affecting the glossopharyngeal and vagus nerves through the left jugular foramen was reported, presenting with hoarseness, dysphagia and uvular deviation (11). Another case of IgG4-related hypertrophic pachymeningitis manifested as glossopharyngeal and vagus nerve palsy with hoarseness, dysphagia, poor gag reflex and soft palate paralysis (12).In addition, hoarseness may result from vocal cord involvement, as demonstrated in a 64-year-old man with IgG4-RD and right vocal cord infiltration (13), or cricoid cartilage ulceration, as seen in a 54-year-old woman with IgG4-RD diagnosed via biopsy (14).Notably, no previous reports describe IgG4-RD concurrently yet exclusively affecting both the carotid artery and vagus nerve. This case expands the recognized clinical spectrum of IgG4-RD and highlights the importance of considering IgG4-RD in the differential diagnosis.

The diagnosis of IgG4-RD requires comprehensive evaluation combining serum IgG4 levels, clinical manifestations, and pathological findings. In this case, the serum IgG4 level was slightly elevated. Pathology showed extensive lymphocyte and plasma cell infiltration with fibrosis, an IgG4+/IgG+ plasma cell ratio slightly greater than 40%, and 12 IgG4-positive plasma cells per high-power field. Storiform fibrosis and obliterative phlebitis were not found. Compared to the typical pathological triad of IgG4-RD (extensive lymphocyte and plasma cell infiltration, storiform fibrosis, obliterative phlebitis), the serum IgG4 level and histopathological features in this case are “borderline.” Because this case is a localized/isolated IgG4-RD, there may be an insufficiency of the classic pathological triad. Although the classic pathological triad is the gold standard, histopathological results partially lacking the triad can still provide useful diagnostic support in an appropriate clinical context. For example, in biopsies from sites such as the meninges or IgG4-related ophthalmic disease(IgG4-ROD), pathology usually shows very little or no storiform fibrosis or obliterative phlebitis (15–17). Furthermore, pathological results are affected by sampling limitations; due to the often focal nature of organ involvement, storiform fibrosis and obliterative phlebitis may be absent in smaller biopsy samples (18). Previous studies have shown that multi-organ involvement is associated with higher serum IgG4 levels (19). The slightly elevated serum IgG4 level in this case may be related to its involvement of a single atypical site. In the face of this situation, comprehensive diagnosis becomes particularly important. This case meets the 2019 American College of Rheumatology/European Alliance of Associations for Rheumatology (ACR/EULAR) classification criteria for IgG4-RD and the 2020 Revised Comprehensive Diagnostic (RCD) criteria, and has ruled out lymphoma, thyroid disease, and Takayasu arteritis. Therefore, the diagnosis is IgG4-RD.

Therapeutic options for IgG4-RD include glucocorticoids, immunosuppressants, and biologic agents, with glucocorticoids being the established first-line therapy. In this case, high-dose glucocorticoid therapy was initially administered but provided limited clinical improvement. Subsequent combination therapy with glucocorticoids and methotrexate also proved ineffective. Ultimately, the addition of tocilizumab resulted in significant mass reduction. While tocilizumab remains less frequently utilized in IgG4-RD management, emerging evidence supports its efficacy. Australian researchers (2022) reported two refractory IgG4-RD cases where tocilizumab induced remission after failed responses to glucocorticoids, methotrexate, and rituximab (20). In 2023, A Japanese case report documented successful tocilizumab treatment in a 66-year-old male patient with concurrent rheumatoid arthritis and IgG4-RD. The patient exhibited marked improvement in arthralgia and parotid/submandibular gland swelling, accompanied by reduced serum IgG4 levels that persisted for over 12 months (21). In the same year, Italian researchers reported two cases of IgG4-RD treated with tocilizumab. The first case involved a 60-year-old woman presenting with IgG4-related aortitis and coronary artery aneurysms, who experienced recurrent myocardial infarction and angina refractory to glucocorticoids and rituximab therapy. Histopathological examination of an aortic wall biopsy revealed IL-6 expression in fibroblasts by *in situ* hybridization. Following tocilizumab administration, inflammatory markers decreased, with complete resolution of aortitis and angina symptoms. The second case involved a 55-year-old male with a retroperitoneal mass accompanied by right hydronephrosis and left pleural thickening. Diagnosis of IgG4-RD was confirmed by pleura biopsy. Following glucocorticoid tapering, disease recurrence occurred. Subsequent rituximab therapy proved ineffective, while tocilizumab treatment ultimately induced disease remission (22) (**Table 1**). Clinical evidence supports the therapeutic efficacy of tocilizumab in IgG4-RD, with IL-6 implicated in disease pathogenesis. Comparative studies have demonstrated elevated serum IL-6 levels in IgG4-RD patients relative to healthy controls, showing positive correlations with both ESR and CRP levels. Immunohistochemical analysis reveals elevated expression of both IL-6 and its receptor (IL-6R) in pathological tissues from retroperitoneal fibrosis and IgG4-related sialadenitis. The IL-6/IL-6R signaling pathway promotes cytokine production by B cells and T cells through fibroblast activation, potentially contributing to the pathogenesis of IgG4-RD (23). A Japanese study demonstrated significant correlations between serum IL-6 levels and inflammatory markers in patients with IgG4-RD (24). These findings indicate that IL-6 likely plays a significant role in the pathogenesis of IgG4-RD. Therefore, tocilizumab - which targets the IL-6 receptor - can inhibit disease progression by blocking the IL-6/IL-6R signaling pathway. In this case, disease remission was observed in the context of combination therapy (glucocorticoids, methotrexate, and tocilizumab), in which tocilizumab likely played a key role by inhibiting the IL−6 signaling pathway. In summary, when glucocorticoids and immunosuppressants show inadequate efficacy in treating IgG4−RD, combination therapy with tocilizumab may be considered.

**Table 1:** Summary of tocilizumab-treated IgG4-RD cases.

Case	Age	Gender	Diagnosis	Involvement of organs	Serological indicators	Previous treatments	Later treatments	Duration of follow-up after tocilizumab administration	Serological and imaging indicators after treatment
Case 1 (22)	60	Female	IgG4-RD	Thoracic aorta, abdominal aorta	IgG4、ESR、CRP were elevated	Glucocorticoid、rituximab	Tocilizumab 162mg/QW subcutaneous injection (SC)	6 months	Complete Remission (CR)
Case 2 (22)	55	Male	IgG4-RD	Left pleura, thoracic aorta, and retroperitoneum	IgG4、ESR、CRP were elevated	Glucocorticoid、rituximab	Tocilizumab 162mg/QW SC	6 months	CR
Case 3 (21)	66	Male	IgG4-RD、rheumatoid arthritis	Bilateral submandibular and parotid glands	IgG4、ESR、CRP were elevated	No	Tocilizumab 162mg/Q2W SC	12 months	Partial Remission (PR)
Case 4 (20)	68	Male	IgG4-RD	Extensive lymph nodes	IgG4、ESR、CRP、ferritin were elevated	Glucocorticoid、rituximab、azathioprine	Tocilizumab 162mg/QW SC	12 months	PR
Case 5 (20)	38	Male	IgG4-RD	constitutional symptoms and anemia.	IgG4、ESR、CRP were elevated	Glucocorticoid、azathioprine、methotrexate、rituximab、mycophenolate mofetil	Tocilizumab 162mg/QW SC	6 months	PR

## Conclusions

This case report describes the first documented occurrence of IgG4-RD with concurrent involvement of both the carotid artery and vagus nerve, presenting with atypical clinical features. These findings expand the recognized clinical spectrum of IgG4-RD and highlight the importance of including this diagnosis in the differential evaluation of patients presenting with atypical symptoms. For patients with IgG4-RD who respond inadequately to conventional glucocorticoids and immunosuppressants, the addition of tocilizumab may be considered as a further therapeutic option.

## Data Availability

The original contributions presented in the study are included in the article/supplementary material. Further inquiries can be directed to the corresponding author.
